# Mitochondrial calcium in cardiac ischemia/reperfusion injury and cardioprotection

**DOI:** 10.1007/s00395-024-01060-2

**Published:** 2024-06-19

**Authors:** Edoardo Bertero, Tudor-Alexandru Popoiu, Christoph Maack

**Affiliations:** 1 Department of Translational Research, Comprehensive Heart Failure Center (CHFC), University Clinic Würzburg, Am Schwarzenberg 15, Haus A15, 97078 Würzburg, Germany; 2 Present Address: Chair of Cardiovascular Disease, Department of Internal Medicine and Specialties (Di.M.I.), University of Genoa, Genoa, Italy; 3 “Victor Babes” University of Medicine and Pharmacy, Timisoara, Romania

**Keywords:** Mitochondria, Myocardial infarction, Ischemia/reperfusion injury, Calcium handling, Reactive oxygen species, Cardiac myocytes

## Abstract

Mitochondrial calcium (Ca^2+^) signals play a central role in cardiac homeostasis and disease. In the healthy heart, mitochondrial Ca^2+^ levels modulate the rate of oxidative metabolism to match the rate of adenosine triphosphate consumption in the cytosol. During ischemia/reperfusion (I/R) injury, pathologically high levels of Ca^2+^ in the mitochondrial matrix trigger the opening of the mitochondrial permeability transition pore, which releases solutes and small proteins from the matrix, causing mitochondrial swelling and ultimately leading to cell death. Pharmacological and genetic approaches to tune mitochondrial Ca^2+^ handling by regulating the activity of the main Ca^2+^ influx and efflux pathways, i.e., the mitochondrial Ca^2+^ uniporter and sodium/Ca^2+^ exchanger, represent promising therapeutic strategies to protect the heart from I/R injury.

## Introduction

Cardioprotection refers to the mechanisms and interventions that protect the heart against various forms of damage, minimizing cardiac myocyte loss and thereby preserving cardiac function [84]. This definition applies in principle to any type of cardiac insult, but cardioprotective strategies were predominantly investigated in the context of myocardial ischemia and infarction. While early reperfusion is crucial to prevent infarct expansion, restoration of blood flow to the ischemic myocardium can be accompanied by reperfusion arrhythmias and reversible contractile dysfunction, known as “myocardial stunning” [18, 56]. The concept of myocardial reperfusion as “a double-edged sword” was first formulated in 1985 by Braunwald and Kloner [20]. Henceforth, studies in this field yielded a detailed understanding of the mechanisms underlying ischemia/reperfusion (I/R) injury, and identified a central role for mitochondria in determining cardiac myocyte fate in this context. While being pivotal to energy transduction via oxidative phosphorylation, mitochondria also act as master regulators of different forms of cell death.

Calcium (Ca^2+^) plays an essential role in cardiac myocyte physiology and disease. In the healthy heart, transient variations in cytosolic Ca^2+^ levels induce contraction and relaxation of cardiac myocytes, and mitochondrial Ca^2+^ signals adapt the rate of mitochondrial oxidative metabolism to the rate of adenosine triphosphate (ATP) turnover in the cytosol. During I/R injury, mitochondrial Ca^2+^ overload is one key mechanism of cardiac myocyte loss by inducing mitochondrial permeability transition. Therefore, therapeutic strategies aimed at modulating intracellular Ca^2+^ movements represent a viable approach to mitigate I/R injury. In this review, we outline the physiological role of mitochondrial Ca^2+^ handling, its alterations during cardiac I/R injury, and how mitochondrial Ca^2+^ signals can be modulated to prevent mitochondrial dysfunction and cardiac myocyte loss upon myocardial reperfusion.

## Physiological role of mitochondrial Ca^2+^ signals

### Mechano-energetic coupling

**Fig. 1:**
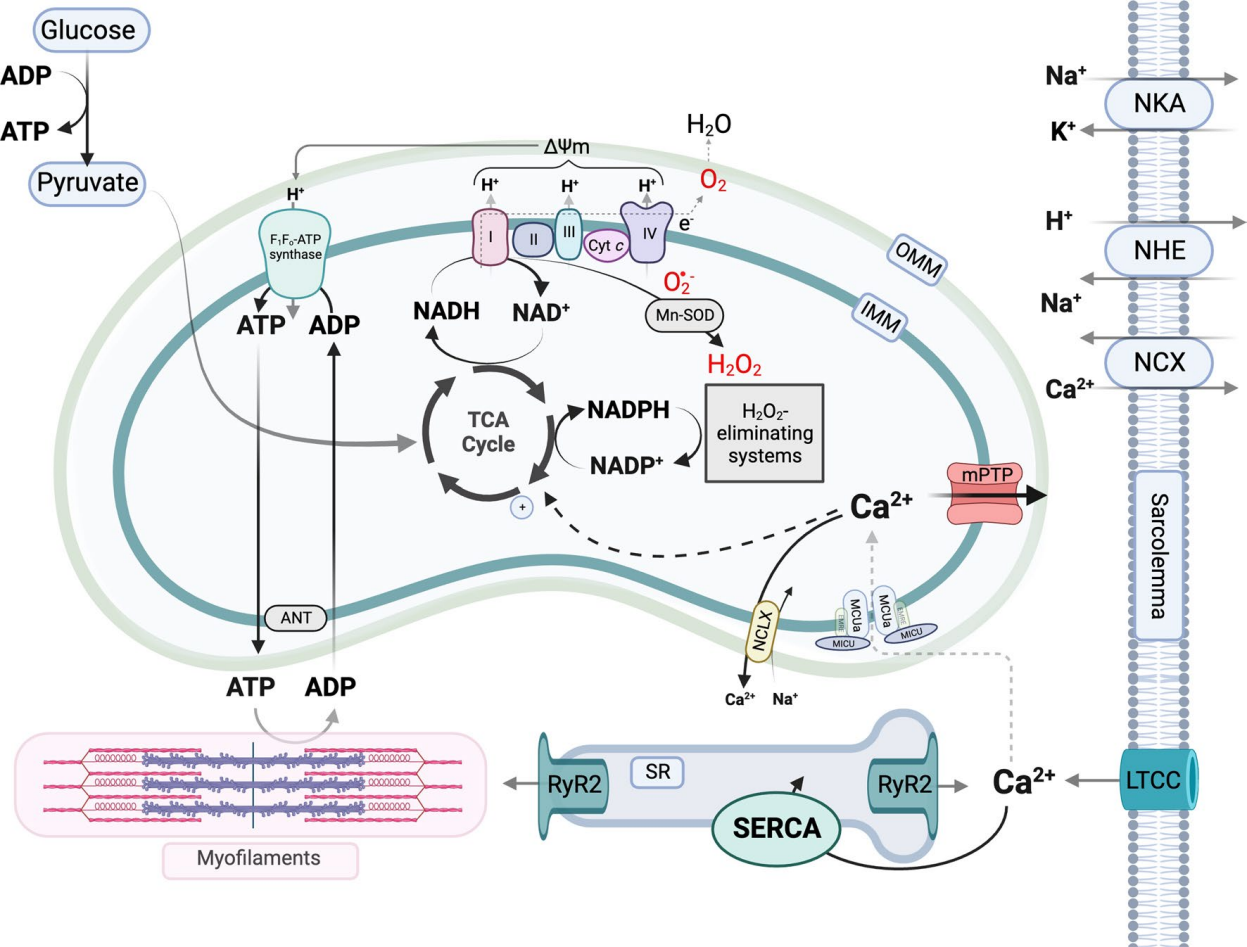
Cardiac mechano-energetic coupling. In the healthy heart, oxidative phosphorylation produces 95% of the adenosine triphosphate (ATP) required to fuel excitation–contraction coupling. ATP phosphorylation is catalyzed by the F_1_/F_o_-ATP synthase, which harnesses the electrochemical gradient produced by translocation of protons (H^+^) across the inner mitochondrial membrane (IMM) by the electron transport chain complexes. In turn, the electron transport chain derives reducing equivalents from the reduced form of nicotinamide adenine dinucleotide (NADH) produced by the tricarboxylic acid (TCA) cycle. Superoxide (^.^O_2_^−^) is a physiological by-product of the respiratory chain activity, and is rapidly dismutated to hydrogen peroxide (H_2_O_2_) by the manganese-dependent superoxide dismutase (Mn-SOD). During elevations in cardiac workload, the increased ATP turnover in the cytosol partially dissipates the mitochondrial membrane potential (ΔΨ_m_) and oxidizes the NADH pool. At the same time, calcium (Ca^2+^) accumulation in the mitochondrial matrix stimulates the TCA cycle dehydrogenases to regenerate NADH and also the reduced form of nicotinamide adenine dinucleotide phosphate (NADPH), which sustain the respiratory chain activity and hydrogen peroxide (H_2_O_2_) elimination, respectively. Also under physiological conditions, transient opening of a low-conductance mitochondrial permeability transition pore (mPTP) in the IMM may operate as an alternative Ca^2+^ efflux pathway in addition to the mitochondrial Na^+^/Ca^2+^ exchanger. Figure created with BioRender.com

Transient variations in cytosolic Ca^2+^ levels ([Ca^2+^]_c_) trigger contraction and relaxation of cardiac myocytes, consuming large amounts of ATP that are continuously regenerated by oxidative phosphorylation in mitochondria and, to a lesser extent, by glycolysis in the cytosol. When increases in cardiac workload accelerate ATP turnover by myofilaments and ion pumps, the increase in adenosine diphosphate (ADP) delivery to mitochondria partially and transiently dissipates the proton motive force (Δμ_H_) as complex V (the F_1_/F_o_-ATP synthase) utilizes the proton gradient across the inner mitochondrial membrane (IMM) to catalyze ADP phosphorylation to ATP. Simultaneously, mitochondrial Ca^2+^ signals adjust the rate of mitochondrial oxidative metabolism by stimulating the activity of three tricarboxylic acid (TCA) cycle enzymes [97], thereby providing more reducing equivalents to the electron transport chain (ETC) to maintain Δμ_H_ [100]. Therefore, ADP and Ca^2+^ induce a “parallel activation” of mitochondrial oxidative metabolism that enables the heart to sustain abrupt changes in contractility (and, consequently, ATP turnover) (Fig. 1).

The reducing equivalents produced from oxidation of nutrients are also used to fuel the activity of antioxidant systems responsible for the elimination of mitochondrial reactive oxygen species (ROS). Namely, the oxidation of malate and isocitrate by the respective dehydrogenases is used to regenerate the reduced form of the cofactor NADPH, which functions as an electron donor for a series of redox reactions that maintain the reduced glutathione (GSH) and thioredoxin (Trx) pools. In turn, GSH and Trx function as reducing agents for the conversion of hydrogen peroxide (H_2_O_2_) to water catalyzed by glutathione peroxidase and peroxiredoxin, respectively. An additional source of mitochondrial NADPH is the nicotinamide nucleotide transhydrogenase (NNT), which uses Δμ_H_ to transfer hydride ion equivalents (H^−^) from NADH to NADPH. Therefore, the TCA cycle is also essential to regenerate the H_2_O_2_-eliminating systems of the mitochondrial matrix [78] (Fig. 1).

Mitochondrial Ca^2+^ signals, therefore, play a central role in cardiac homeostasis, as they allow the heart to respond to sudden changes in workload without incurring in an energetic crisis or inducing oxidative stress. The ability of mitochondrial oxidative metabolism to swiftly adapt to elevations in ATP turnover, coined mechano-energetic coupling, is impaired in acute and chronic cardiovascular disorders, including I/R injury and heart failure (reviewed in [14]).

### The mitochondrial Ca^2+^ uniporter (MCU)

The molecular identity of the channel(s) mediating Ca^2+^ uptake from the cytosol to the mitochondrial matrix has remained elusive until the early 2010s, when the mitochondrial Ca^2+^ uniporter (MCU) pore-forming subunit, MCUa, was identified [11, 132]. It became apparent that the MCU has a complex architecture and, besides a tetramer of MCUa subunits forming the channel pore, it comprises at least four other core components: the essential MCU regulatory element, EMRE, and the regulatory proteins MICU1, MICU2, and MICU3 [36]. EMRE is a transmembrane protein that is essential for Ca^2+^ uptake via the MCU in metazoans [125]. MICU1, 2, and 3 regulate the MCU current depending on Ca^2+^ levels in the intermembrane space, which they sense via their EF-hand domains. The mechanisms of MICU-mediated regulation of Ca^2+^ flux through the MCU is still under investigation. There is consensus that MICU1 accounts for the cooperative activation of the MCU at high [Ca^2+^]_c_, whereas at low [Ca^2+^]_c_, MICU1 physically occludes the pore [122], thus preventing uncontrolled influx of Ca^2+^ in the mitochondrial matrix at the physiological [Ca^2+^]_c_ ranges of 100 to 500 nM (so-called gatekeeper function). In line with this model, MICU1 deletion increases mitochondrial Ca^2+^ levels at low [Ca^2+^]_c_, but blunts the rapid MCU-mediated increase in matrix Ca^2+^ when mitochondria are exposed to high Ca^2+^ [74, 90, 110].

Because of the low Ca^2+^ affinity of the uniporter, mitochondrial Ca^2+^ uptake via the MCU is made possible by the close proximity of mitochondria to the points of Ca^2+^ release from the sarcoplasmic reticulum (SR). The apposition of the outer mitochondrial membrane and the SR membrane creates specialized microdomains where spatially localized elevations of [Ca^2+^]_c_ to micromolar levels overcome the threshold required for Ca^2+^ uptake via the MCU, thus driving Ca^2+^ influx to the mitochondrial matrix [42]. Several proteins have been implicated in the physical tethering of mitochondria and the SR, including mitofusin (MFN) 1 and 2 and FUN domain containing protein 1 [137]. MFN1 and MFN2 play a primary role in modulating the structural and functional juxtaposition between the SR and mitochondria, as well as the interorganellar exchange of Ca^2+^ [21, 24].

MFN1 and MFN2 were initially discovered as mediators of mitochondrial fusion [23], the process by which two mitochondria merge into a single, larger mitochondrion. This allows exchange of mitochondrial DNA and other components to compensate for mitochondrial damage. Conversely, mitochondrial fission is the process where one mitochondrion divides into two separate mitochondria. Mitochondrial fission ensures apportioning of mitochondria during cell division and segregation of damaged mitochondrial components for elimination by mitophagy [54]. There is a bidirectional interaction between SR-mitochondria Ca^2+^ movements and mitochondrial dynamics. Mitochondrial morphology, influenced by the balance between fission and fusion, regulates mitochondrial Ca^2+^ uptake [82]. Conversely, changes in mitochondrial Ca^2+^ uptake can also affect fission and fusion dynamics [81]. However, these cross-regulatory mechanisms were mainly investigated in cell systems, and evidence of their role in the intact heart is lacking.

### Mitochondrial Ca^2+^ efflux

The best characterized mitochondrial Ca^2+^ efflux pathway is the mitochondrial sodium (Na^+^)/Ca^2+^ exchanger (NCLX) [22, 113]. The Na^+^ gradient across the IMM is the primary determinant of NCLX activity, and thereby elevations in cytosolic sodium levels ([Na^+^]_c_) accelerate NCLX-mediated Ca^2+^ extrusion from mitochondria [12, 28]. One Na^+^-independent mechanism of Ca^2+^ efflux was recently described [8], but its role in cardiac myocytes has not been investigated thus far. The slower kinetics of mitochondrial Ca^2+^ efflux compared with MCU-dependent influx account for the progressive increase in matrix Ca^2+^ that stimulates the TCA cycle dehydrogenases during elevations in heart rate and contractility, which prevents oxidation of mitochondrial pyridine nucleotides [96]. The exact quantities of mitochondrial Ca^2+^ are not completely resolved; it is unclear whether mitochondria significantly buffer [Ca^2+^]_c_ and whether matrix Ca^2+^ oscillates on a beat-to-beat basis, paralleling changes in [Ca^2+^]_c_, or integrates cytosolic Ca^2+^ transients in a frequency-dependent manner [14].

In conclusion, mitochondrial Ca^2+^ handling plays a relevant physiological role in matching ATP supply with demand in cardiac myocytes. Ca^2+^ levels in the mitochondrial matrix are finely tuned by the balance between Ca^2+^ uptake via the MCU and Ca^2+^ extrusion via the NCLX, driven by the Na^+^ gradient across the IMM. The structural and functional interaction between SR and mitochondria is pivotal to the transmission of Ca^2+^ signals to the mitochondrial matrix.

## Mitochondrial Ca^2+^ overload and permeability transition in I/R injury

### Consequences of ischemia on cardiac myocyte ion handling

Alterations in mitochondrial Ca^2+^ handling have important consequences on cardiac myocyte function and viability. In chronic heart failure, insufficient mitochondrial Ca^2+^ accumulation causes bioenergetic mismatch and oxidative stress during transitions of cardiac workload [78, 79]. The opposite alteration, i.e., mitochondrial Ca^2+^ overload, is a major driver of cardiac myocyte loss in the context of I/R injury and therefore, represents a potential target for cardioprotection.

**Fig. 2:**
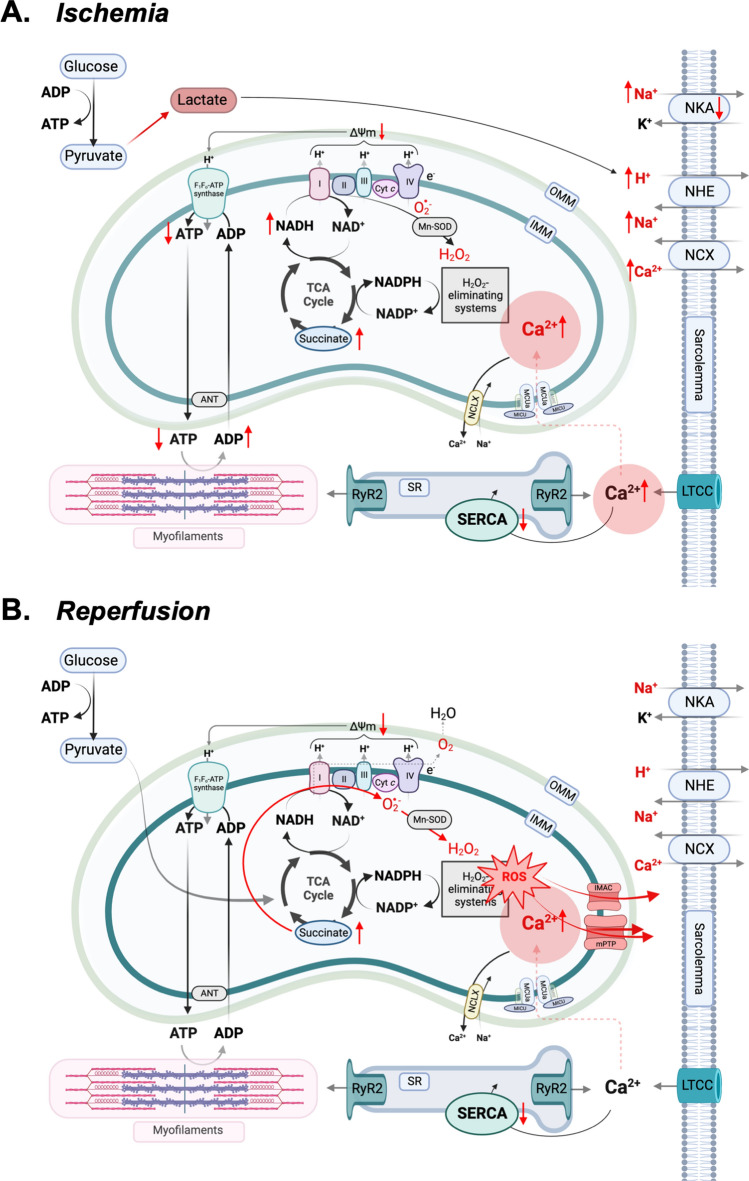
Mechanisms of cardiac ischemia/reperfusion injury. Panel A. Ischemia. During ischemia, the lack of oxygen (O_2_) abruptly halts oxidative phosphorylation and induces a metabolic switch to anaerobic glycolysis, which increases lactate production and induces intracellular acidosis. The reduced availability of ATP stops cardiac myocyte contraction and relaxation and alters intracellular ion concentrations. Reduced activity of the sodium (Na^+^)/potassium (K^+^) ATPase, together with the increased activity of the Na^+^/H^+^ exchanger due to intracellular acidosis, induce Na^+^ and Ca^2+^ accumulation in the cytosol. The increase in calcium (Ca^2+^) levels is transmitted to the mitochondrial matrix via the mitochondrial Ca^2+^ uniporter. Mitochondrial Ca^2+^ accumulation progressively dissipates the mitochondrial membrane potential (ΔΨ_m_), which can ultimately trigger mitochondrial permeability transition and necrotic cell death. Panel B. Reperfusion***.*** When blood flow is restored, the activity of the respiratory chain resumes, but succinate accumulated during ischemia fuels superoxide (O_2_^−^) production at complex I via reverse electron transport. The massive production of mitochondrial reactive oxygen species (ROS), together with mitochondrial Ca^2+^ accumulation and partial restoration of intracellular pH, triggers irreversible opening of the large-conductance mitochondrial permeability transition pore (mPTP), which allows release of ions, solutes and ROS from the mitochondrial matrix. ROS release from the mPTP and other redox-sensitive channels, such as the inner mitochondrial anion channel (IMAC), can induce ROS production from neighboring mitochondria, a process coined ROS-induced ROS release. Figure created with BioRender.com

During myocardial ischemia, the reduction in oxygen (O_2_) and nutrient supply to the myocardium halts mitochondrial oxidative metabolism. The arrest of oxidative phosphorylation leads to a rapid decline in cellular ATP and phosphocreatine below the levels required to sustain cardiac myocyte contraction and relaxation. Subsequently, the activity of plasma membrane ATP-dependent Na^+^ pumps decreases, resulting in an increase in [Na^+^]_c_ and potassium (K^+^) efflux from the cell. A compensatory increase in anaerobic glycolysis leads to H^+^ accumulation in the cytosol [130], which decreases myofilament Ca^2+^ affinity [35] and leads to further Na^+^ influx via the sarcolemmal Na^+^/H^+^ exchanger (NHE) [49]. The increase in [Na^+^]_c_ shifts the driving force of the sarcolemmal Na^+^/Ca^2+^ exchanger (NCX) toward reverse mode, thus favoring Ca^2+^ influx from the extracellular space [66]. This, together with Ca^2+^ entry via L-type Ca^2+^ channels and impaired SR Ca^2+^ reuptake due to reduced SR Ca^2+^ ATPase (SERCA) activity, contributes to [Ca^2+^]_c_ elevation, which is transmitted to the mitochondrial matrix (Fig. 2). Ultimately, prolonged depletion of ATP generates a hyperosmolar environment that promotes cell swelling and leads to disruption of the protein synthetic apparatus, causing cell death.

### Consequences of reperfusion on cardiac myocyte ion handling

If blood flow is restored before the ischemic myocardium is irreversibly damaged, the recovery of sarcolemmal Na^+^/K^+^ ATPase and NHE activity rapidly restores intracellular Na^+^ and K^+^ levels and pH, respectively. However, reperfusion can result, paradoxically, in the death of cardiac myocytes that are not otherwise irreversibly injured by ischemia, a process coined I/R injury. The underlying mechanism is in part related to the increased influx of leukocytes and aberrant immune responses in the ischemic tissue upon reperfusion [34]. In addition, cell death during reperfusion can be triggered by cardiac myocyte-specific processes that result from the abrupt restoration of O_2_ availability.

Mitochondrial Ca^2+^ overload is considered a major contributor to irreversible mitochondrial damage and subsequent cardiac myocyte death during I/R injury [38, 48]. Uncontrolled Ca^2+^ release from the SR leads to abnormal [Ca^2+^]_c_ oscillation and mitochondrial Ca^2+^ overload in the early stages of reperfusion [1, 124]. There are two potential pathways of Ca^2+^ influx in the matrix from the cytosol during I/R injury, i.e., the MCU and/or the NCLX operating in reverse mode [7], but their relative contribution is still unresolved. The mechanisms linking mitochondrial Ca^2+^ overload to cell death have been debated, but there is now substantial evidence indicating that excess matrix Ca^2+^, together with oxidative stress occurring during reperfusion, triggers the opening of a large pore in the IMM, coined the mitochondrial permeability transition pore (mPTP), which leads to the release of proteins and solutes up to ~ 1.5 kDa from the matrix.

### The mitochondrial permeability transition pore

It has long been known that a non-selective increase in permeability occurs in mitochondrial membrane after exposure to Ca^2+^, but this was considered a consequence of membrane damage until the seminal studies by Hunter and Haworth in the 1970s first proposed the concept of mitochondrial permeability transition [52, 62]. The existence of the mPTP was supported by the subsequent identification of a large-conductance channel in the IMM [77] and the observation that permeability transition can be inhibited by cyclosporine A (CsA), which argues against a non-specific disruption of the IMM accounting for permeability transition [29, 40]. The inhibitory effect exerted by CsA was shown to be dependent on a matrix peptidyl prolyl cis–trans isomerase, cyclophilin D (CypD) [45, 134], whose genetic ablation desensitizes the mPTP to Ca^2+^ [9, 104].

The molecular composition of the mPTP is still under investigation. The adenine nucleotide translocator (ANT) was first proposed as a mPTP component based on the modulatory effects of its inhibitors on permeability transition [61]. However, genetic ablation of ANT does not abolish mPTP opening, although it increases by tenfold the Ca^2+^ threshold for permeability transition [80]. Another candidate mPTP component is the F_1_/F_o_-ATP synthase, which can generate Ca^2+^-induced high-conductance currents when reconstituted in lipid bilayers [44]. A unifying and now widely accepted model posits that permeability transition is mediated by two types of pores, one with low and one with high conductance. The former opens transiently and operates to re-equilibrate ion concentrations across the IMM under physiological conditions, whereas the latter, by allowing the leakage of larger solutes, induces important alterations on mitochondrial structure that can ultimately lead to cell death (for a recent review see [19]). ANT is a candidate component of the low-conductance mPTP [106], whereas F_1_/F_o_-ATP synthase dimers might form the high-conductance channel within their F_o_ subunits in response to structural rearrangements induced by Ca^2+^ binding to the F_1_ subunit [43, 118]. CypD, the target of CsA, functions as a chaperone protein that promotes the conformational modification of the F_1_/F_o_-ATP synthase by binding to its regulatory oligomycin sensitivity-conferring protein (OSCP) subunit [43].

The threshold for mitochondrial Ca^2+^ required to trigger permeability transition is modulated by multiple factors. In particular, mPTP opening is promoted by low ADP levels, dissipation of Δμ_H_, and production of ROS, and antagonized by magnesium (Mg^2+^) and pH values < 7 [19]. Despite the increase in mitochondrial Ca^2+^, permeability transition is inhibited by high ADP levels and the acidic cellular milieu induced by ischemia [3]. Therefore, the majority of mPTP-mediated cardiac myocyte loss occurs during the early stage of reperfusion, when ATP levels increase and ion pumps reprise their activity to restore cellular pH [32, 46]. In addition, mPTP opening is promoted by the increase in mitochondrial ROS production occurring during reperfusion, discussed in the next section.

One additional mechanism of mPTP regulation involves dynamin-related protein 1 (Drp1), an essential mediator of mitochondrial fission. During mitochondrial fission, Drp1 is recruited to the outer mitochondrial membrane, where it forms a ring-like structure that constricts the dividing mitochondrion [87]. Although mitochondrial fission and Drp1 are essential for normal cardiac development and homeostasis [65], enhanced mitochondrial fission driven by Drp1 recruitment to mitochondria exacerbates cardiac myocyte loss during I/R injury. Indeed, acute pharmacological or genetic inhibition of Drp1 protects the heart from I/R injury by preventing mPTP opening [108]. The mechanism of cardioprotection afforded by acute Drp1 ablation might be explained by increased Ca^2+^ retention capacity and decreased ROS production of larger, undivided mitochondria compared with fragmented mitochondria generated by excessive fission [117]. For a more comprehensive discussion on the role of mitochondrial dynamics in cardiac homeostasis and cardioprotection, we refer the reader to another review of this series [54].

The prolonged and widespread opening of the high-conductance mPTP during reperfusion injury aggravates the redistribution of ions and solutes and causes osmotic swelling of the mitochondrial matrix. Altogether, the arrest of oxidative metabolism, collapse of membrane potential, and irreversible mitochondrial and cellular alterations lead to cell necrosis. Of note, mitochondrial permeability transition can also induce apoptosis mediated by the release of proapoptotic factors normally sequestered within mitochondria, such as cytochrome *c*. Since apoptosis is an ATP-dependent process, ATP availability is the key factor determining the mode of cell death after mitochondrial permeability transition [75].

## Mitochondrial reactive oxygen species in I/R injury

Mitochondrial ROS are a physiological by-product of aerobic metabolism. The incomplete reduction of O_2_ gives rise to superoxide (O_2_^.−^), an extremely reactive and short-lived form of ROS that can cause cellular damage by subtracting electrons to lipids, nucleic acids, or proteins. The respiratory chain, and namely complex I and complex III, are considered major cellular sources of superoxide [102]. To dispose of superoxide, mitochondria are equipped with micromolar concentrations of manganese-dependent superoxide dismutase (Mn-SOD), which dismutates superoxide into H_2_O_2_. As described above, H_2_O_2_ is converted to water by matrix peroxidases, i.e., peroxiredoxin and glutathione peroxidases, which derive their reducing equivalents from NADPH and thereby, from the TCA cycle.

Mitochondrial production of ROS plays a central role in the pathophysiology of I/R injury [57]. The burst of mitochondrial superoxide occurring at the onset of reperfusion was initially considered a non-specific consequence of the restored availability of O_2_, but there is now robust evidence indicating that reverse electron transport (RET) at complex I of the respiratory chain is the primary source of superoxide during reperfusion [25]. The mechanistic underpinnings of this process and its role in I/R injury are discussed in detail in another review of this series [120]. Here, we outline the concept of redox-optimized ROS balance, a unifying framework that explains how the overflow of ROS is determined by the redox environment of the cell [5] and ROS-induced ROS release, a self-amplifying process that can exacerbate cardiac myocyte loss and induce arrhythmias during I/R injury [143].

### Redox-optimized ROS balance

**Fig. 3:**
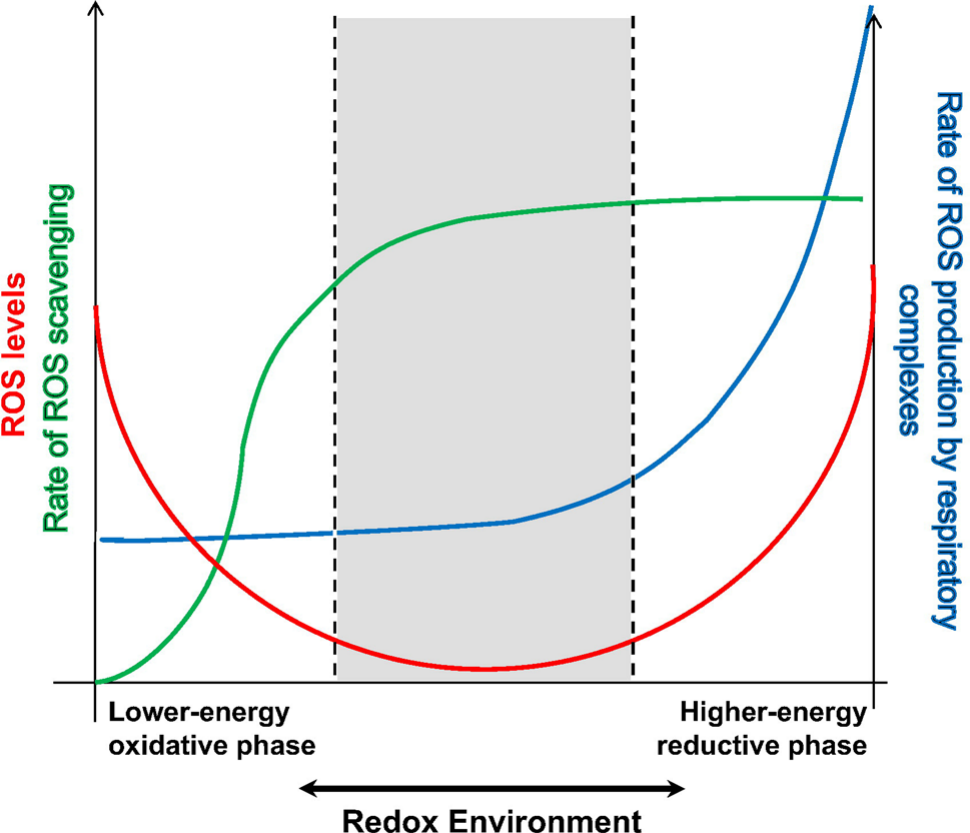
Redox-optimized ROS balance. The graph illustrates the relationship between the redox environment of cardiac myocytes and the emission of reactive oxygen species (ROS). ROS emission occurs when the intracellular and/or intramitochondrial environments are either highly reduced (right side of the figure) or highly oxidized (left). Under physiological conditions, the cell and mitochondria operate at intermediate redox state (gray-shaded area), at which ROS production is controlled by the antioxidant systems. Reproduced with permission from [5]. Copyright Elsevier

Initial observations in isolated mitochondria demonstrated that ROS production is maximized under conditions of little electron flow, high mitochondrial membrane potential (ΔΨ_m_), and a fully reduced NADH pool. In this context, ROS production can be alleviated by “mild” uncoupling [101]. These observations stand at odds with experiments in intact cardiac myocytes, in which mitochondrial uncoupling increases ROS emission. The concept of redox-optimized ROS balance solves this apparent contradiction by proposing that the extent of ROS overflow is determined by the overall intracellular redox environment, which includes the redox couples involved in electron transport, i.e., NADH/NAD^+^ and the ubiquinone pool, and those involved in ROS elimination, i.e., NADPH/NADP and the glutathione pool. Oxidative stress occurs at either extreme of redox potential, that is, when the intramitochondrial environment is either highly reduced or highly oxidized [5] (Fig. 3). Mitochondria normally operate at intermediate redox states, characterized by low ROS production at the respiratory chain and sufficient levels of reduced NADPH to prevent H_2_O_2_ emission.

Redox-optimized ROS balance also explains the increase in ROS production observed during hypoxia, a seeming paradox when considering the dependence of superoxide production on O_2_ concentration. According to this concept, hypoxic cells and mitochondria operate in the right arm of the redox extreme curve (Fig. 3), implying that they exhibit highly reduced redox potentials and augmented superoxide production due to the low electron flow [5]. However, since ROS formation at the ETC also depends linearly on the availability of O_2_ per se [10], superoxide formation during hypoxia is still limited despite a highly reduced redox state of the ETC. At reperfusion (and thereby, reoxygenation), the increase in O_2_ meets a highly reduced respiratory chain, provoking a “burst” of ROS production and release in the very first seconds and minutes of reperfusion, which levels off once the redox state of the respiratory chain becomes more oxidized due to the reinstallment of electron flux for ATP production. These processes add (and are part of) the concept of the burst-like release of ROS due to reverse electron transfer provoked by succinate accumulation during ischemia. Furthermore, a depletion of glutathione via multidrug resistance protein 1 (MRP-1) during ischemia depletes the antioxidative defense and therefore, adds to the burst of ROS during reperfusion [64].

Mitochondria, however, can also be pushed toward the more oxidized extreme of the redox spectrum under pathological conditions. In systolic heart failure, insufficient Ca^2+^-dependent stimulation of the TCA cycle chronically depletes mitochondrial antioxidant systems, leading to oxidative stress that drives maladaptive remodeling and arrhythmias [78, 91]. Furthermore, an acute oxidation of the mitochondrial NAD(P)H pool can be induced by the opening of redox-sensitive ion channels in the IMM, including the mPTP, which is the basis for ROS-induced ROS release.

### ROS-induced ROS release

Mitochondria permeability transition can exacerbate mitochondrial ROS production via a process coined ROS-induced ROS release [144]. This process involves the ROS-dependent induction of redox-sensitive ion channels within the IMM, such as the mPTP [143], inner mitochondrial anion channel (IMAC) [2, 4], and mitochondrial ATP-dependent K^+^ channels (mK_ATP_) [31, 76] of nearby or neighboring mitochondria. Opening of these channels dissipates ΔΨ_m_ and oxidizes the NADH and NADPH pools, consequently diminishing antioxidant defenses and causing ROS generation. This ROS-induced ROS release, coupled with IMAC activation, fosters synchronized oscillations of ΔΨ_m_, establishing “metabolic sinks” that render specific myocardial regions electrically inactive, thereby contributing to re-entrant arrhythmias during cardiac I/R [2]. It is unclear whether the IMAC coincides with the low-conductance, reversible mPTP described above, whose opening occurs at lower levels of oxidative stress and can be reversed, or whether it represents a distinct entity. In addition, ROS-induced ROS release has been observed through interactions between distinct ROS sources. ROS derived from NADPH oxidase 2 can elevate mitochondrial ROS [30, 76, 95] and vice versa, mitochondrial ROS can exacerbate NADPH oxidase-induced ROS production [83].

In conclusion, although mitochondrial ROS production is increased also under hypoxic conditions, the largest quantity of ROS is produced during reperfusion, primarily via reverse electron transport at complex I. Besides the ETC, there are other important sources of ROS in mitochondria that have been implicated in I/R injury, such as monoamine oxidases (discussed in another review of this series [73]), NADPH oxidases [141], and p66^shc^ [119]. Oxidative stress, in concert with mitochondrial Ca^2+^ overload, can trigger mitochondrial permeability transition and ROS-induced ROS release, exacerbating cellular damage and creating a substrate for ventricular arrhythmias during I/R injury.

## Role of mitochondrial Ca^2+^ in ischemic conditioning

**Fig. 4:**
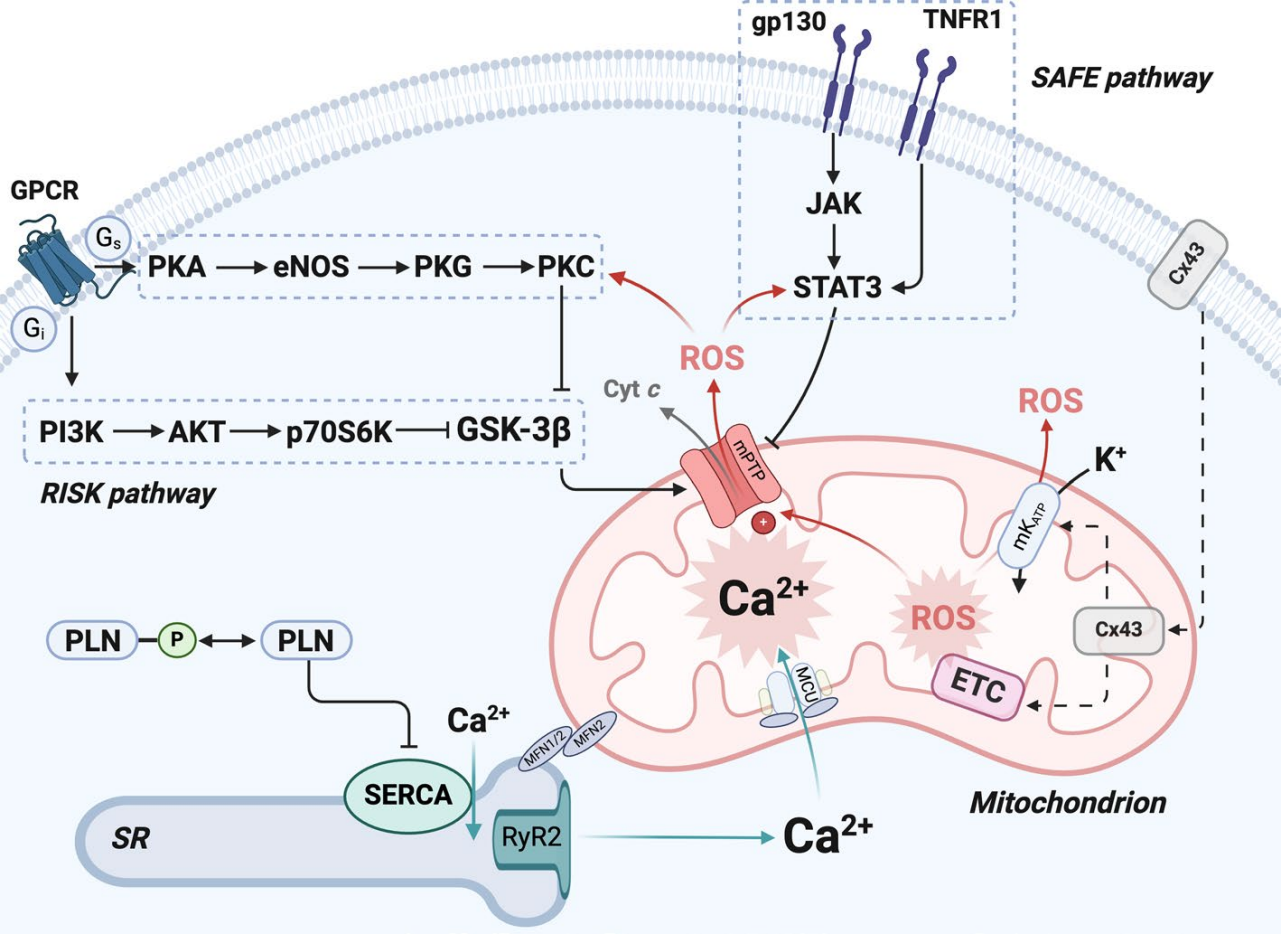
Role of mitochondria in ischemic conditioning. The main signaling pathways activated during ischemic conditioning include the reperfusion injury salvage kinase (RISK) pathway, the survival activating factor enhancement (SAFE) pathway, and a pathway involving activation of endothelial nitric oxide synthase (eNOS), protein kinase G (PKG) and protein kinase C (PKC), which mainly converge on mitochondria as the end effector of cardioprotection. Opening of the mitochondrial permeability transition pore (mPTP), which is triggered by mitochondrial Ca^2+^ overload and reactive oxygen species (ROS) production, is a major cause of cardiac myocyte loss during ischemia/reperfusion injury. Modulation of mitochondrial permeability transition is considered a central mediator of cardioprotection induced by ischemic conditioning. Intracellular signaling pathways activated by intermittent cycles of ischemia and reperfusion reduce susceptibility to mPTP opening via multiple mechanisms, including direct mPTP inhibition, mitochondrial translocation of connexin 43 (Cx43), activation of mitochondrial potassium (K^+^) uptake via the mitochondrial ATP-dependent K^+^ channel (mK_ATP_), and reduced formation of reactive oxygen species (ROS). In addition, modulation of Ca^2+^ exchange between the sarcoplasmic reticulum (SR) and mitochondria contributes to the cardioprotection afforded by ischemic conditioning. Namely, decreased Ca^2+^ release via ryanodine receptor type 2 (RyR2) and delayed phospholamban (PLN) phosphorylation might prevent exaggerate SR Ca^2+^ release and consequent mitochondrial Ca^2+^ overload during reperfusion. *Cyt c* cytochrome *c*, *GPCR* G protein-coupled receptor, *GSK-3β* glycogen synthase kinase-3β, *JAK* Janus kinase, *MCU* mitochondrial calcium uniporter, *MFN1/2* mitofusin ½, *PKA* protein kinase A, *SERCA* sarcoplasmic reticulum Ca^2+^ ATPase, *SR*, sarcoplasmic reticulum, *STAT3* signal transducer and activator of transcription 3; *TNFR* tumor necrosis factor receptor. Figure created with BioRender.com

Ischemic conditioning refers to the activation of a cardioprotective program induced by repeated brief episodes of coronary occlusion and reperfusion preceding or following the prolonged insult. Ischemic conditioning in the heart was first described in a series of seminal studies by the group of Keith Reimer, who showed that brief, intermittent cycles of I/R delay the rate of ATP depletion and markedly reduce infarct size in hearts subsequently subjected to sustained ischemia [103]. Subsequent studies demonstrated that a protective effect against prolonged ischemia is achieved also when intermittent ischemia is applied during early reperfusion, a process coined ischemic *post*-conditioning [142], and when they involve extracardiac tissues, known as *remote* conditioning [41]. The mechanisms underlying ischemic pre-, post-, and remote conditioning (IPC, POC, and RIC, respectively) are manifold [55], and involve the release of soluble mediators including adenosine, cytokines, and neurohormones. In turn, these mediators bind to specific receptors expressed by cardiac myocytes and activate intracellular signaling cascades, including the reperfusion injury salvage kinase (RISK) pathway, survival activating factor enhancement (SAFE) pathway, and a pathway involving endothelial nitric oxide synthase, protein kinase G and protein kinase C. These pathways primarily converge on mitochondria as the end effectors of cardioprotection [55]. Namely, modulation of mPTP opening is considered the key mediator of cardioprotection induced by ischemic conditioning. Other mitochondrial ion channels, i.e., mK_ATP_ and connexin 43 (Cx43), are also known to modulate susceptibility to myocardial I/R injury [51, 55] (Fig. 4).

### Mitochondrial ATP-dependent K^+^ channels

ATP-dependent K^+^ channels (K_ATP_) are present on both the sarcolemma and IMM and their activation is regulated by the ATP/ADP ratio [67]. When ATP levels drop, opening of sarcolemmal K_ATP_ functions as an “energy-sparing” mechanism by shortening action potential duration, consequently reducing cytosolic Ca^2+^ entry and depressing contractility [39]. Initially, ischemic conditioning was attributed to sarcolemmal K_ATP_ based on the observation that K_ATP_ openers mimicked IPC, while K_ATP_ inhibitors abolished it [139]. However, later studies showed limited correlation between action potential shortening and cardioprotection conferred by K_ATP_ inhibitors [47], K_ATP_ opener protection in unstimulated cells [89], and negligible protection from agents targeting only sarcolemmal K_ATP_ [126]. These findings shifted the focus to mitochondrial K_ATP_ (mK_ATP_) as mediators of IPC. Since K^+^ influx osmotically drives water in the mitochondrial matrix, mK_ATP_ activity is a key regulator of mitochondrial volume. Multiple mechanisms were proposed to explain the cardioprotective effect of mK_ATP_ opening. First, K^+^ influx partially dissipates Δμ_H_, thereby reducing the driving force for mitochondrial Ca^2+^ uptake [26, 92]. In addition, mK_ATP_ opening might modulate mitochondrial permeability transition indirectly, by activating cardioprotective signaling pathways. For instance, the mK_ATP_ opener diazoxide and the K^+^ ionophore valinomycin elicit PKCε-dependent phosphorylation of the mPTP by inducing mild ROS release from the matrix [27].

A limitation of these studies is that they are based on pharmacological inducers or inhibitors of mK_ATP_ opening, while investigations employing in vivo genetic interventions on the channel are hindered by the elusive molecular identity of mK_ATP_. CCDC51, a protein with previously unknown function, was identified to interact with the known mK_ATP_ regulator ATP Binding Cassette protein 8 (ABCB8) to form a channel with mK_ATP_-like properties [109]. Accordingly, genetic ablation of CCDC51 protects the heart against I/R injury [109]. An alternative and paradigm-shifting model posits that the F_1_/F_o_-ATP synthase (complex V of the ETC) can use both H^+^ and K^+^ to synthesize ATP and represents the primary way for K^+^ to enter mitochondria [70]. By mediating this previously unrecognized K^+^ flux, the F_1_/F_o_-ATP synthase might also function as a recruitable mK_ATP_ channel, whereby mitochondrial K^+^ influx and consequent increase in matrix volume result in desensitization of the mPTP [71]. Further expanding this model, the same authors discovered that two members of the survival proteins family, Bcl-xL and Mcl-1, modulate chemo-mechanical efficiency of the F_1_/F_o_-ATP synthase to function as mK_ATP_ [71]. Altogether, further studies in vivo are warranted to confirm this suggested role of the ATP synthase as mK_ATP_.

### Mitochondrial connexin 43

Connexin 43 (Cx43) is a major component of gap junctions, i.e., intercellular channels that allow diffusion of ions and small molecules between adjacent cardiac myocytes. The cardioprotective effect of IPC, but not POC, is abolished in mouse hearts with a heterozygous deletion of *Cx43* gene [58, 127]. The effect of IPC is also lost when isolated Cx43-deficient cardiac myocytes are exposed to simulated ischemia (i.e., hypoxia and acidosis) in vitro, suggesting that cell–cell communication mediated by Cx43 is not the sole mechanism for its protective effect [88]. Subsequent studies linked Cx43-dependent cardioprotection to its localization to subsarcolemmal mitochondria, where its expression is increased by IPC [15, 17]. Mechanistically, IPC induces Cx43 translocation to the IMM via the translocase of the outer membrane (TOM) complex [123]. However, the precise mechanisms by which mitochondrial translocation of Cx43 protects the heart remain unclear. Cx43 interacts with several mitochondrial proteins [33] and modulates critical mitochondrial functions, including complex I respiration [16], ROS formation [53], and K^+^ and Ca^2+^ uptake [99, 131], all of which potentially contribute to its cardioprotective activity. An additional layer of complexity arises from Cx43 phosphorylation by at least three kinases: mitogen-activated protein kinase (MAPK), protein kinase C (PKC), and casein kinase 1 (CK1), the latter of which is determinant for the cardioprotection by IPC, in the absence of any effect on ROS formation and mPTP opening in isolated mitochondria [60]. Altogether, these findings suggest a potentially multifaceted role of Cx43 in cardioprotection, which partly depends on its effects on mitochondrial ROS and Ca^2+^ and is modulated by CK1 phosphorylation via yet unknown mechanisms.

### Role of mitochondrial Ca^2+^ and permeability transition in ischemic conditioning

The mPTP is considered a crucial effector of ischemic conditioning. The prevailing view posits that ischemic conditioning is mediated by transient (low-conductance) opening of the mPTP inducing a controlled release of Ca^2+^ from mitochondria, which alleviates Ca^2+^ overload and prevents sustained mPTP opening during prolonged ischemia [50]. Indeed, inhibitors of mPTP opening abolish the protection associated with both IPC and POC [6, 50]. Elucidating the precise mechanisms by which intracellular signaling cascades elicited by intermittent ischemia modulate mPTP opening remains an active area of research. One potential link between cardioprotective signaling and mPTP modulation is glycogen synthase kinase-3β (GSK-3β), which acts as a downstream target for multiple kinases and whose phosphorylation limits mitochondrial permeability transition [72]. Furthermore, activation of the Janus kinase 2 (JAK2)/signal transducer and activator of transcription 3 (STAT3) axis, which can be induced by a moderate increase in ROS production, mediates the cardioprotective effect of POC by preserving mitochondrial respiration and increasing mitochondrial Ca^2+^ retention capacity [59, 135, 136] (Fig. 4).

Modulation of Ca^2+^ exchange between the SR and mitochondria contributes to the cardioprotection afforded by ischemic conditioning. One mechanism implicated in IPC-mediated cardioprotection is a decrease in Ca^2+^ release from the SR via the type 2 ryanodine receptor (RyR2) upon reperfusion [133], which lowers mitochondrial Ca^2+^ levels and potentially also explains the reduction in reperfusion-induced arrhythmias observed with IPC [128]. Moreover, at the onset of reperfusion, rapid phosphorylation of phospholamban relieves its inhibition of SERCA contributing to SR and, consequently, mitochondrial Ca^2+^ overload. By delaying phospholamban phosphorylation, POC might promote cellular Ca^2+^ extrusion via the NCX and prevent exaggerate SR Ca^2+^ release [68] (Fig. 4).

Altogether, modulation of mPTP opening emerges as the end effector of cardioprotection induced by ischemic conditioning. As detailed in the following section, strategies that attenuate mitochondrial Ca^2+^ overload effectively limit mPTP opening and subsequent cardiomyocyte death during I/R injury. Conversely, mitochondrial ROS production has a Janus-faced role. While excessive ROS generation during reperfusion is a well-established mediator of cellular damage, a moderate ROS emission through mK_ATP_ and mPTP appears to be required for the cardioprotective effects of ischemic conditioning [50, 129]. Several key questions remain to be definitively addressed, e.g., the precise threshold of mitochondrial Ca^2+^ uptake that triggers irreversible overload and the specific molecular links between cardioprotective signaling cascades involved in ischemic conditioning and the modulation of mPTP opening.

## Modulation of mitochondrial Ca^2+^ handling for cardioprotection

### Pharmacological and genetic inhibition of the MCU

The central role played by mitochondrial Ca^2+^ overload in triggering permeability transition and consequent cardiac myocyte loss implies that preventing mitochondrial Ca^2+^ uptake represents a viable therapeutic approach to mitigate myocardial damage during reperfusion. Early studies using ruthenium red to block mitochondrial Ca^2+^ uptake during reperfusion showed reduced myocardial O_2_ consumption, preserved contractile function, and decreased myocardial damage in the isolated rat heart ex vivo [13]. Ruthenium red, however, inhibits the enzymatic activity of several other proteins involved in excitation–contraction coupling and Ca^2+^ handling, including SERCA and RyR2 [145], which could partly account for the observed benefit. Subsequent studies using the more specific MCU inhibitor ruthenium 360 (Ru360) confirmed the protective effect of blocking mitochondrial Ca^2+^ uptake on myocardial performance in isolated rat hearts [69]. Conversely, the MCU activator spermine blunts the protective effect of ischemic conditioning [135, 140].

The discovery of the molecular identity of the MCU complex paved the way to a series of studies that investigated the effects of genetic silencing of the MCU pore and regulatory components on I/R injury. Surprisingly, global constitutive knockout of the MCUa gene (*Mcua*-/-) in mice did not affect viability and cardiac function, but impaired exercise capacity [115]. Cardiac mitochondria isolated from *Mcua*-/- mice did not exhibit rapid, high-capacity mitochondrial Ca^2+^ uptake nor mPTP opening after prolonged exposure to high Ca^2+^ [115]. Nevertheless, *Mcua*-/- mice were not protected against cardiac I/R injury, and could not be protected by treatment with CsA [115]. In stark contrast, mice with conditional and cardiac myocyte-specific deletion of *Mcua* during adult life exhibited a 50% reduction in infarct size after I/R injury [85, 94]. Taken together, *Mcua* deletion before birth does not protect against I/R injury, whereas inducible *Mcua* silencing has a strong cardioprotective effect. Another potential approach to decrease MCUa levels is to induce degradation of *Mcua* mRNA with miR-25, which is protective in vitro but was never tested in vivo [114].

### MCUb overexpression

MCUb is a paralog of MCUa and functions as a negative regulator of MCU flux by forming hetero-oligomers with MCUa, thus altering the stoichiometry and function of the uniporter [121]. Both constitutive and inducible cardiac myocyte-specific MCUb overexpression confer protection against ischemic injury [63, 86]. Under physiological conditions, the MCU complex of cardiac myocytes does not comprise MCUb subunits. Cardiac stressors such as ischemia induce MCUb expression and thereby decrease Ca^2+^ uptake via incorporation of MCUb in the MCU complex. However, MCUb induction occurs too late after the onset of ischemia to protect the heart from acute I/R injury [86]. Accordingly, mice lacking MCUb do not show differences in acute cardiac injury following I/R, but exhibit substantially worse left ventricular dilatation and contractile dysfunction 2 to 4 weeks after the ischemic event [63]. Altogether, the results of these studies indicate that, although MCUb upregulation occurs too late to mitigate acute cardiac myocyte loss during I/R, it represents an adaptive mechanism that prevents further cardiac myocyte death and scar expansion by reducing the sensitivity of mitochondria to elevated [Ca^2+^]_c_ [86].

### MICU1 modulation

MICU1 exerts an important regulatory role on MCU flux. Physiologically, the MICU1/MCU ratio determines tissue-specific differences in the [Ca^2+^]_c_ threshold for Ca^2+^ uptake [111]. In myocardial samples from patients with heart failure, MICU1 levels and the MICU1/MCU ratio are increased [112], which might explain the differences in MCU current measured in mitoplasts (i.e., mitochondria stripped of the outer membrane) isolated from myocardial samples of heart failure patients [98]. On the other hand, mitochondrial localization of MICU1 mediated by the translocase of the outer membrane 70 (TOM70) is impaired after myocardial I/R, which decreases the MICU1/MCU ratio [138]. MICU1 silencing aggravates mitochondrial Ca^2+^ overload during I/R injury, thereby worsening apoptosis, cardiac remodeling, and contractile dysfunction [138]. On these grounds, it has been proposed that the gatekeeping function exerted by MICU1 on MCU flux can be exploited to protect the heart from I/R injury.

### NCLX

One additional strategy to prevent Ca^2+^-induced cell death is to accelerate Ca^2+^ extrusion from the mitochondrial matrix by increasing NCLX activity. Cardiac-specific overexpression of the NCLX reduces superoxide production, prevents cardiac myocyte loss and preserves contractile function in mice subjected to I/R [93]. Vice versa, cardiac myocyte-specific deletion of NCLX gene in the adult mouse heart leads to mitochondrial Ca^2+^ overload and mPTP opening that result in severe systolic dysfunction and death within days after ablation of mitochondrial Ca^2+^ efflux [93]. Cardiac dysfunction and lethality induced by NCLX silencing are rescued by genetic deletion of CypD, which underscores the importance of mPTP opening as a mediator of cell death induced by mitochondrial Ca^2+^ overload [93].

### Mitofusin 2

In principle, modulation of mitochondrial Ca^2+^ signals can also be achieved by regulating the structural and functional proximity of mitochondria and the SR. However, the role of proteins mediating SR-mitochondria tethering in cardiac physiology and pathology remains incompletely resolved. In particular, it is debated whether MFN2 functions as a tether or a spacer in the SR-mitochondria contact sites [37, 105]. In cell lines and mice in which MFN2 is genetically deleted, mitochondrial Ca^2+^ uptake and the Ca^2+^-mediated bioenergetic adaptation of cardiac myocytes are blunted, and mice are protected against Ca^2+^ overload-induced mPTP opening, indicating that the SR-mitochondria juxtaposition is decreased by MFN2 ablation [24, 105, 116]. This concept was subsequently challenged by Filadi and colleagues, who proposed that MFN2 ablation in fact potentiates interorganellar communication, thereby sensitizing MFN2-deficient cells to Ca^2+^ overload-mediated cell death [37]. These conflicting results might be explained by differences in genetic manipulation (inducible vs constitutive knockout) and cellular/animal models. An additional factor of complexity is the different effect of acute vs chronic modulation of SR/mitochondria tethering. The results of a recent study suggest that while acute changes in tethering may cause dysfunction, chronic enhancement of SR/mitochondria contact sites from early life induces an adaptive remodeling of the organelles that improves the resilience to stressors associated with mitochondrial Ca^2+^ overload, including adrenergic stimulation and I/R injury [107].

## Conclusions

Taken together, mitochondrial Ca^2+^ handling is pivotal to the regulation of mitochondrial redox state, which impacts ATP production and mitochondrial antioxidative capacity. While in systolic heart failure, decreased mitochondrial Ca^2+^ uptake and steady-state Ca^2+^ accumulation hampers TCA cycle activation, which leads to oxidation of pyridine nucleotides and elevated ROS emission, excessive mitochondrial uptake during I/R injury provokes mPTP opening and cell death. Since most of the evidence for the role of the MCU and NCLX on cardiac physiology and pathology derives from genetic mouse models, and the physiological role and regulation of mitochondrial steady-state Ca^2+^ is very different between mice and human (due to a tenfold difference in resting heart rate), a note of caution is raised when interpreting results from genetic mouse studies. Nevertheless, considering the opposing defects in terms of mitochondrial Ca^2+^ uptake in systolic heart failure versus I/R injury, targeting mitochondrial Ca^2+^ handling therapeutically can be a double-edged sword, but may be successful when tailored to the respective pathological conditions. Additional research is warranted to better understand the fine-tuning of mitochondrial Ca^2+^ handling, its alterations in different pathological conditions, but also species (and potentially, sex-) dependent differences to further establish such therapies.
